# Synthesis and crystal structure analysis of 1-ethyl-1,3-di­hydro-2*H*-benzo[*d*]imidazole-2-thione

**DOI:** 10.1107/S2056989025000519

**Published:** 2025-01-28

**Authors:** Atash V. Gurbanov, Firudin I. Guseinov, Aida I. Samigullina, Tuncer Hökelek, Khudayar I. Hasanov, Tahir A. Javadzade, Alebel N. Belay

**Affiliations:** a Excellence Center, Baku State University, Z. Xalilov Str. 23, AZ 1148 Baku, Azerbaijan; b Kosygin State University of Russia, 117997 Moscow, Russian Federation; c N. D. Zelinsky Institute of Organic Chemistry, Russian Academy of Sciences, 119991 Moscow, Russian Federation; d Hacettepe University, Department of Physics, 06800 Beytepe-Ankara, Türkiye; e Western Caspian University, Istiglaliyyat Str. 31, AZ 1001 Baku, Azerbaijan; f Azerbaijan Medical University, Scientific Research Centre (SRC), A. Kasumzade Str. 14, AZ 1022 Baku, Azerbaijan; g Department of Chemistry and Chemical Engineering, Khazar University, Mahzati Str. 41, AZ 1096 Baku, Azerbaijan; h Department of Chemistry, Bahir Dar University, PO Box 79, Bahir Dar, Ethiopia; University of Kentucky, USA

**Keywords:** noncovalent inter­actions, hydrogen bonding, crystal structure

## Abstract

The asymmetric unit of the title compound, C_9_H_10_N_2_S, contains two crystallographically independent, almost planar, mol­ecules. In the crystal, inter­mole­cular N—H⋯S hydrogen bonds link the mol­ecules into pseudocentrosymmetric dimers, enclosing *R*_2_^2^(8) ring motifs. There are mutual π–π inter­actions between the five- and six-membered rings of each independent mol­ecule in the chosen asymmetric unit, with ring centroid-to-centroid distances of 3.6685 (12) and 3.7062 (12) Å. A weak C—H⋯π(ring) inter­action is also observed. The N—H⋯S hydrogen bonds, the π–π inter­actions and the weak C—H⋯π(ring) inter­action are effective in the stabilization of the crystal structure. The structure was refined as an inversion twin with a component occupancy ratio of 0.546 (15):0.454 (16).

## Abstract 2

Mol­ecules of 1-ethyl-1,3-di­hydro-2*H*-benzo[*d*]imidazole-2-thione are almost planar. In the crystal, inter­molecular N—H⋯S hydrogen bonds link the mol­ecules into pseudocentrosymmetric dimers. N—H⋯S hydrogen bonds, π–π inter­actions and a weak C—H⋯π(ring) inter­action are effective in the stabilization of the crystal structure.

## 1. Chemical context

Benzimidazoles are defined as a class of heterocyclic aromatic organic compounds characterized by a benzene ring fused to an imidazole ring at specific positions, exhibiting both acidic and weakly basic properties (Gaba *et al.*, 2014 ▸). Benzimidazole and its derivatives have attracted considerable inter­est in recent years for their versatile properties in chemistry and pharmacology (Akhtar *et al.*, 2017 ▸; Khalilov *et al.*, 2024 ▸). They are used as auxilliary ligands in the synthesis of coordination compounds (Jlassi *et al.*, 2014 ▸; Mizar *et al.*, 2012 ▸). Thus, benzimidazole compounds have been an inter­esting resource for researchers for more than a century (Guseinov *et al.*, 2006 ▸, 2017 ▸, 2020 ▸; Rzayev & Khalilov, 2024 ▸). For instance, 2-mer­captobenzimidazole was successfully built into zeolitic imidazolate framework-8 on graphene oxide nanosheets and then embedded into an ep­oxy coating to prepare a composite coating with pH-responsive and self-healing performance (Li *et al.*, 2021 ▸). The attachment of noncovalent halogen-bond donor or acceptor site(s) to benzimidazole can be used as a synthetic strategy in the design of catalysts, materials and drugs (Ma *et al.*, 2021 ▸; Mahmoudi *et al.*, 2017*a* ▸,*b* ▸; Shixaliyev *et al.*, 2014 ▸). Herein, we have synthesized 1-ethyl-1,3-di­hydro-2*H*-benzo[*d*]imidazole-2-thione by the reaction of *N*^1^-ethyl­benzene-1,2-di­amine with carbon di­sulfide in the presence of pyridine (see Scheme[Chem scheme1]) and studied its mol­ecular and crystal structures.
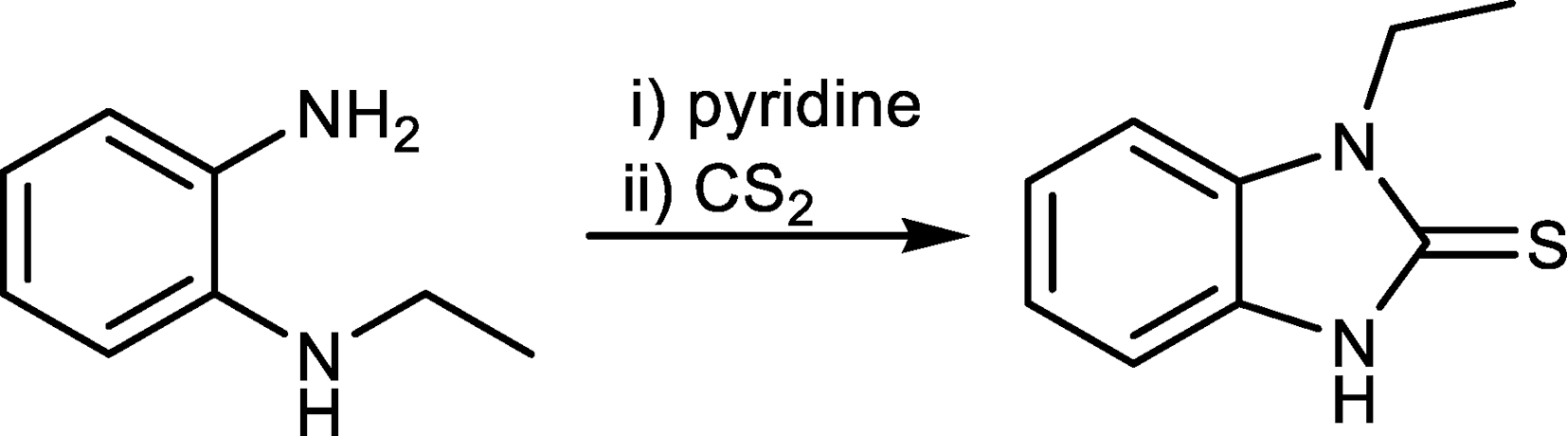


## 2. Structural commentary

**Figure 1:**
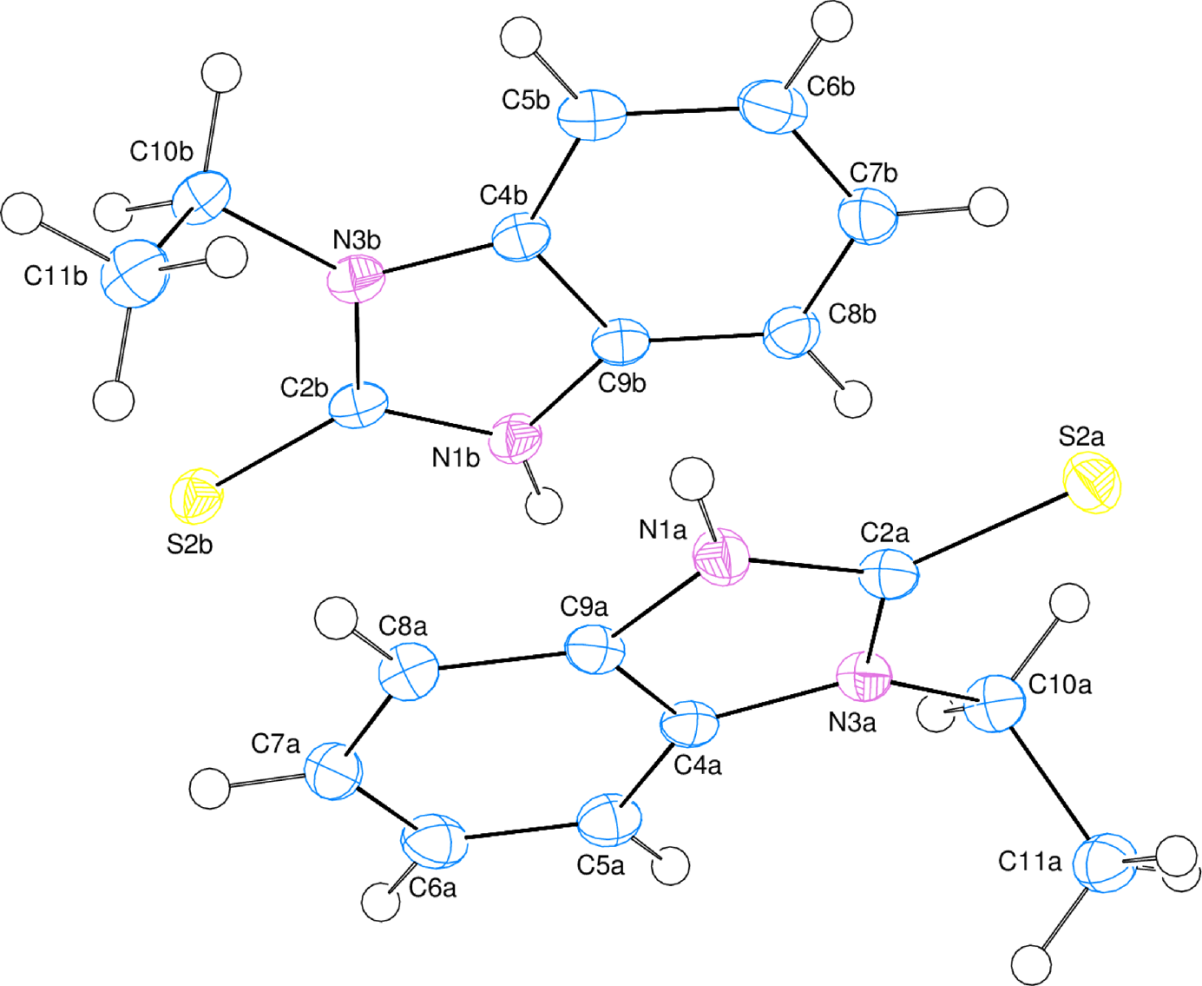
The title mol­ecules with the atom-numbering scheme and 50% probability ellipsoids.

The asymmetric unit of the title structure contains two crystallographically independent mol­ecules (Fig. 1 ▸). The planar *A* (atoms C4a–C9a), *B* (N1a/N3a/C2a/C4a/C9a), *C* (C4b–C9b) and *D* (N1b/N3b/C2b/C4b/C9b) rings are oriented at dihedral angles of *A*/*B* = 0.75 (9)° and *C*/*D* = 1.64 (12)°. Thus, they are almost coplanar. On the other hand, atoms S2a/C10a and S2b/C10b are 0.0061 (3)/−0.1824 (15) and 0.0864 (4)/−0.0278 (15) Å away from the best least-squares planes of the *B* and *D* rings, respectively. Thus, they are almost coplanar with the corresponding ring planes. The orientations of the ethyl groups relative to the benzimidazole fused rings may be described by the torsion angles C2a—N3a—C10a—C11a = 91.4 (2)°, C4a—N3a—C10a—C11a = −98.0 (2)°, C2b—N3b—C10b—C11b = 94.4 (2)° and C4b–N3b—C10b—C11b = −84.5 (2)°. There no unusual bond distances or inter­bond angles in the mol­ecules. The structure was refined as an inversion twin with a component occupancy ratio of 0.546 (15):0.454 (16).

## 3. Supra­molecular features

**Figure 2:**
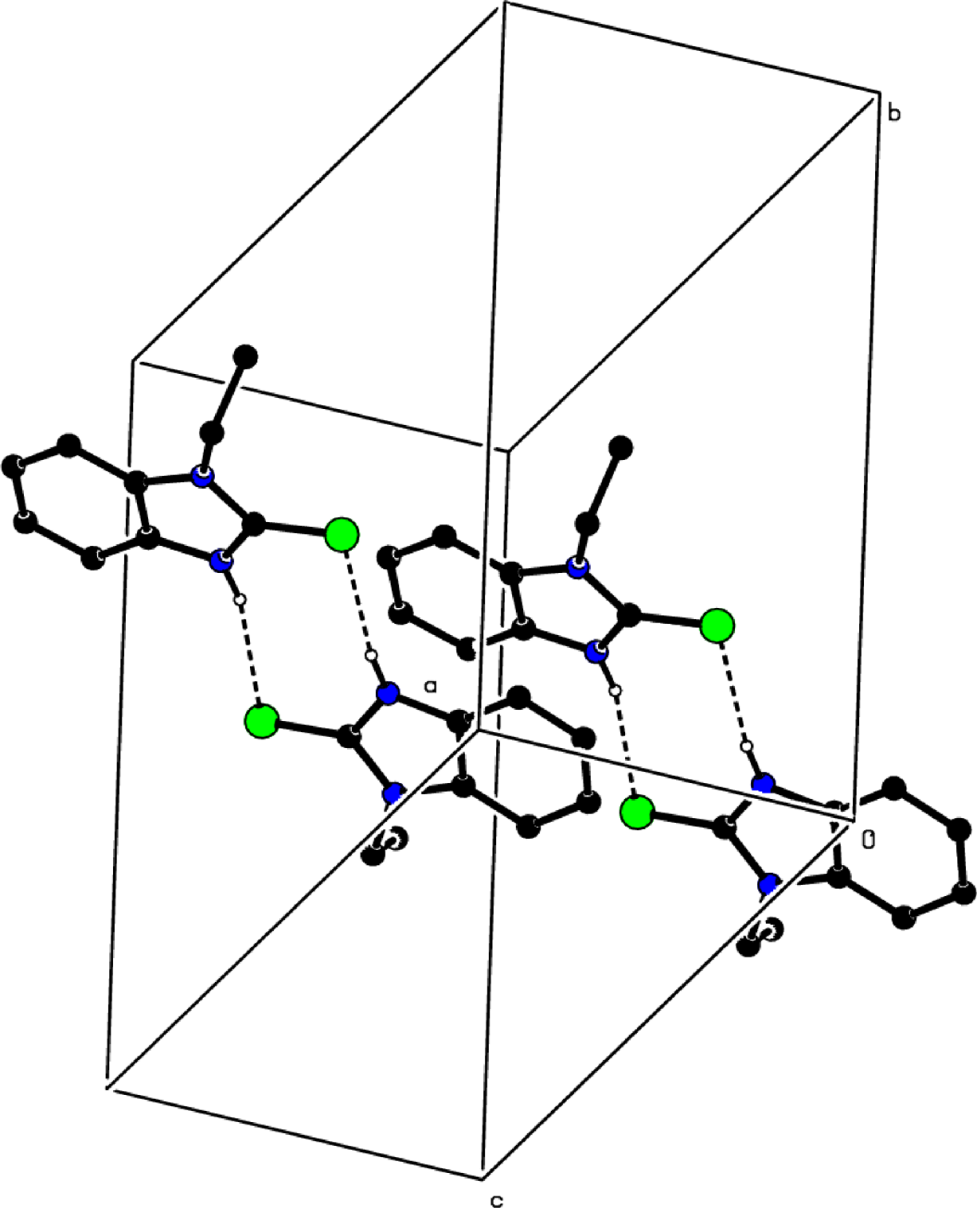
A partial packing diagram. Inter­molecular N—H⋯S hydrogen bonds are shown as dashed lines.

In the crystal, inter­molecular N—H⋯S hydrogen bonds (Table 1 ▸) link the mol­ecules into pseudocentrosymmetric dimers, enclosing 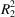
(8) ring motifs, where the mol­ecules are stacked along the *a*-axis direction (Fig. 2 ▸). There are π–π inter­actions between the *B* and *C* rings, and between the *A* and *D* rings, with centroid-to-centroid distances of 3.6685 (12) [dihedral = 11.02 (10)° and slippage = 0.413 Å] and 3.7062 (12) Å [dihedral = 11.26 (11)° and slippage = 0.405 Å], respectively. A weak C—H⋯π(ring) inter­action is also observed (Table 1 ▸). The N—H⋯S hydrogen bonds, the π–π inter­actions and the weak C—H⋯π(ring) inter­action are effective in the stabilization of the crystal structure.

## 4. Synthesis and crystallization

CS_2_ (228 mg, 3.00 mmol) was added to a solution of *N*^1^-ethyl­benzene-1,2-di­amine (136 mg, 1.00 mmol) in pyridine (10 ml) and the resulting solution refluxed for 8 h. The reaction mixture was then evaporated in a vacuum and the residue crystallized from ethanol. The title compound was obtained in the form of orange crystals (yield: 146 mg, 82%; m.p. 120–122 °C) which were soluble in methanol, ethanol and dimethyl sulfoxide (DMSO). Analysis calculated (%) for C_9_H_10_N_2_S: C 60.64, H 5.65, N 15.72; found: C 60.61, H 5.65, N 15.69. ^1^H NMR (300 MHz, DMSO-*d*_6_): δ 1.19 (3H, CH_3_), 3.82 (2H, CH_2_), 6.83–7.08 (4H, Ar-H), 10.87 (1H, NH). ^13^C NMR (75 MHz, DMSO-*d*_6_): δ 13.8, 34.8, 107.9, 108.8, 120.4, 120.6, 128.3, 129.8, 153.9.

## 5. Refinement

Crystal data, data collection and structure refinement details are summarized in Table 2 ▸. The NH hydrogens were located in a difference Fourier map and refined freely. The C-bound H-atom positions were calculated geometrically at distances of 0.95 (for aromatic CH), 0.99 (for CH_2_) and 0.98 Å (for CH_3_), and refined using a riding model by applying the constraint *U*_iso_(H) = *kU*_eq_(C), where *k* = 1.5 for methyl H atoms and 1.2 for the other H atoms. The title compound was refined as an inversion twin with component ratio occupancies of 0.546 (15):0.454 (16).

## Supplementary Material

Crystal structure: contains datablock(s) I, global. DOI: 10.1107/S2056989025000519/pk2714sup1.cif

Structure factors: contains datablock(s) I. DOI: 10.1107/S2056989025000519/pk2714Isup2.hkl

Supporting information file. DOI: 10.1107/S2056989025000519/pk2714Isup3.cml

CCDC reference: 2418168

Additional supporting information:  crystallographic information; 3D view; checkCIF report

## Figures and Tables

**Table 1:** Hydrogen-bond geometry (Å, °) *Cg*3 is the centroid of the C4*A*⋯C9*A* ring.

*D*—H⋯*A*	*D*—H	H⋯*A*	*D*⋯*A*	*D*—H⋯*A*
N1*B*—H1*B*⋯S2*A*^i^	0.84 (3)	2.49 (3)	3.3258 (18)	171 (3)
N1*A*—H1*A*⋯S2*B*^ii^	0.88 (3)	2.41 (3)	3.2654 (18)	165 (3)
C6*B*—H6*B*⋯*Cg*3^iii^	0.95	2.85	3.6470 (17)	143

Symmetry codes: (i) 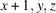; (ii) 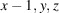; (iii) 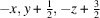.

**Table 2:** Experimental details

Crystal data	
Chemical formula	C_9_H_10_N_2_S
*M*_r_	178.25
Crystal system, space group	Orthorhombic, *P*2_1_2_1_2_1_
Temperature (K)	100
*a*, *b*, *c* (Å)	7.4512 (5), 14.77990 (12), 15.89120 (11)
*V* (Å^3^)	1750.06 (12)
*Z*	8
Radiation type	Cu *K*α
μ (mm^−1^)	2.80
Crystal size (mm)	0.41 × 0.24 × 0.17

Data collection	
Diffractometer	Rigaku XtaLAB Synergy Dualflex diffractometer with a HyPix detector
Absorption correction	Gaussian (*CrysAlis PRO*; Rigaku OD, 2023 ▸)
*T*_min_, *T*_max_	0.405, 1.000
No. of measured, independent and observed [*I* > 2σ(*I*)] reflections	23801, 3799, 3779
*R*_int_	0.035
(sin θ/λ)_max_ (Å^−1^)	0.638

Refinement	
*R*[*F*^2^ > 2σ(*F*^2^)], *wR*(*F*^2^), *S*	0.026, 0.069, 1.06
No. of reflections	3799
No. of parameters	229
H-atom treatment	H atoms treated by a mixture of independent and constrained refinement
Δρ_max_, Δρ_min_ (e Å^−3^)	0.25, −0.21
Absolute structure	Refined as an inversion twin.
Absolute structure parameter	0.454 (16)

Computer programs: *CrysAlis PRO* (Rigaku OD, 2023 ▸), *SHELXT2014* (Sheldrick, 2015*a* ▸), *SHELXL2018* (Sheldrick, 2015*b* ▸) and *OLEX2* (Dolomanov *et al.*, 2009 ▸).

## supplementary crystallographic information

### 1-Ethyl-1,3-dihydro-2H-benzo[d]imidazole-2-thione Crystal data

**Table:**

C_9_H_10_N_2_S	*D*_x_ = 1.353 Mg m^−^^3^
*M**_r_* = 178.25	Cu *K*α radiation, λ = 1.54184 Å
Orthorhombic, *P*2_1_2_1_2_1_	Cell parameters from 18990 reflections
*a* = 7.4512 (5) Å	θ = 4.1–79.0°
*b* = 14.77990 (12) Å	µ = 2.80 mm^−^^1^
*c* = 15.89120 (11) Å	*T* = 100 K
*V* = 1750.06 (12) Å^3^	Block, colorless
*Z* = 8	0.41 × 0.24 × 0.17 mm
*F*(000) = 752	

### 1-Ethyl-1,3-dihydro-2H-benzo[d]imidazole-2-thione Data collection

**Table:**

Rigaku XtaLAB Synergy Dualflex diffractometer with a HyPix detector	3799 independent reflections
Radiation source: micro-focus sealed X-ray tube, PhotonJet (Cu) X-ray Source	3779 reflections with *I* > 2σ(*I*)
Mirror monochromator	*R*_int_ = 0.035
Detector resolution: 10.0000 pixels mm^-1^	θ_max_ = 79.5°, θ_min_ = 4.1°
ω scans	*h* = −9→9
Absorption correction: gaussian (*CrysAlis PRO*; Rigaku OD, 2023)	*k* = −18→18
*T*_min_ = 0.405, *T*_max_ = 1.000	*l* = −15→20
23801 measured reflections	

### 1-Ethyl-1,3-dihydro-2H-benzo[d]imidazole-2-thione Refinement

**Table:**

Refinement on *F*^2^	Hydrogen site location: mixed
Least-squares matrix: full	H atoms treated by a mixture of independent and constrained refinement
*R*[*F*^2^ > 2σ(*F*^2^)] = 0.026	*w* = 1/[σ^2^(*F*_o_^2^) + (0.0359*P*)^2^ + 0.5853*P*] where *P* = (*F*_o_^2^ + 2*F*_c_^2^)/3
*wR*(*F*^2^) = 0.069	(Δ/σ)_max_ = 0.001
*S* = 1.06	Δρ_max_ = 0.25 e Å^−^^3^
3799 reflections	Δρ_min_ = −0.21 e Å^−^^3^
229 parameters	Extinction correction: SHELXL2018 (Sheldrick 2015b), Fc^*^=kFc[1+0.001xFc^2^λ^3^/sin(2θ)]^-1/4^
0 restraints	Extinction coefficient: 0.0010 (2)
Primary atom site location: dual	Absolute structure: Refined as an inversion twin.
Secondary atom site location: difference Fourier map	Absolute structure parameter: 0.454 (16)

### 1-Ethyl-1,3-dihydro-2H-benzo[d]imidazole-2-thione Special details

**Table:**

Geometry. All esds (except the esd in the dihedral angle between two l.s. planes) are estimated using the full covariance matrix. The cell esds are taken into account individually in the estimation of esds in distances, angles and torsion angles; correlations between esds in cell parameters are only used when they are defined by crystal symmetry. An approximate (isotropic) treatment of cell esds is used for estimating esds involving l.s. planes.
Refinement. Refined as a 2-component inversion twin.

### 1-Ethyl-1,3-dihydro-2H-benzo[d]imidazole-2-thione Fractional atomic coordinates and isotropic or equivalent isotropic displacement parameters (Å^2^)

**Table:**

	*x*	*y*	*z*	*U*_iso_*/*U*_eq_	
S2A	−0.22204 (7)	0.21363 (3)	0.89250 (3)	0.02146 (13)	
N1A	−0.0479 (2)	0.19722 (11)	0.74247 (10)	0.0187 (3)	
N3A	0.1123 (2)	0.15428 (11)	0.85066 (10)	0.0173 (3)	
C2A	−0.0506 (3)	0.18841 (13)	0.82739 (12)	0.0182 (4)	
C4A	0.2181 (3)	0.14132 (12)	0.77941 (12)	0.0177 (4)	
C5A	0.3909 (3)	0.10730 (14)	0.76978 (14)	0.0218 (4)	
H5A	0.459639	0.087262	0.816552	0.026*	
C6A	0.4579 (3)	0.10412 (14)	0.68818 (15)	0.0250 (4)	
H6A	0.575684	0.081670	0.678910	0.030*	
C7A	0.3556 (3)	0.13326 (14)	0.61921 (14)	0.0234 (4)	
H7A	0.405983	0.130237	0.564370	0.028*	
C8A	0.1826 (3)	0.16641 (14)	0.62913 (13)	0.0218 (4)	
H8A	0.112969	0.185807	0.582421	0.026*	
C9A	0.1164 (3)	0.16980 (13)	0.71074 (13)	0.0180 (4)	
C10A	0.1735 (3)	0.14556 (14)	0.93791 (13)	0.0212 (4)	
H10A	0.117518	0.193980	0.972037	0.025*	
H10B	0.305098	0.154553	0.939713	0.025*	
C11A	0.1290 (3)	0.05472 (14)	0.97697 (14)	0.0247 (4)	
H11A	0.182860	0.006293	0.943261	0.037*	
H11B	−0.001560	0.046798	0.978545	0.037*	
H11C	0.176860	0.052327	1.034369	0.037*	
S2B	0.71248 (7)	0.32708 (3)	0.61994 (3)	0.02249 (13)	
N1B	0.5206 (2)	0.33699 (12)	0.76571 (11)	0.0201 (3)	
N3B	0.3941 (2)	0.40686 (11)	0.65996 (11)	0.0188 (3)	
C2B	0.5411 (3)	0.35762 (13)	0.68320 (13)	0.0189 (4)	
C4B	0.2768 (3)	0.41420 (13)	0.72778 (12)	0.0189 (4)	
C5B	0.1079 (3)	0.45342 (14)	0.73544 (14)	0.0229 (4)	
H5B	0.051416	0.482733	0.689295	0.028*	
C6B	0.0258 (3)	0.44772 (14)	0.81367 (15)	0.0259 (4)	
H6B	−0.089939	0.473475	0.821187	0.031*	
C7B	0.1094 (3)	0.40496 (14)	0.88166 (14)	0.0247 (4)	
H7B	0.049496	0.402837	0.934396	0.030*	
C8B	0.2777 (3)	0.36553 (13)	0.87409 (13)	0.0218 (4)	
H8B	0.334784	0.336867	0.920438	0.026*	
C9B	0.3587 (3)	0.37001 (13)	0.79548 (13)	0.0191 (4)	
C10B	0.3654 (3)	0.44431 (14)	0.57602 (13)	0.0205 (4)	
H10C	0.483111	0.454711	0.548751	0.025*	
H10D	0.304031	0.503466	0.581024	0.025*	
C11B	0.2538 (3)	0.38201 (15)	0.52124 (13)	0.0262 (5)	
H11D	0.238632	0.409231	0.465462	0.039*	
H11E	0.135862	0.372960	0.547203	0.039*	
H11F	0.314744	0.323560	0.515652	0.039*	
H1B	0.596 (4)	0.308 (2)	0.7946 (19)	0.034 (8)*	
H1A	−0.130 (4)	0.225 (2)	0.7125 (19)	0.032 (7)*	

### 1-Ethyl-1,3-dihydro-2H-benzo[d]imidazole-2-thione Atomic displacement parameters (Å^2^)

**Table:**

	*U*^11^	*U*^22^	*U*^33^	*U*^12^	*U*^13^	*U*^23^
S2A	0.0218 (2)	0.0243 (2)	0.0183 (2)	0.00626 (19)	0.00212 (18)	0.00069 (17)
N1A	0.0183 (8)	0.0205 (8)	0.0173 (8)	0.0042 (7)	−0.0001 (6)	0.0007 (6)
N3A	0.0185 (8)	0.0153 (7)	0.0181 (8)	0.0005 (6)	−0.0010 (6)	−0.0004 (6)
C2A	0.0201 (9)	0.0145 (8)	0.0198 (9)	0.0009 (7)	−0.0014 (8)	−0.0011 (7)
C4A	0.0185 (9)	0.0128 (8)	0.0217 (9)	−0.0010 (7)	−0.0005 (8)	−0.0008 (7)
C5A	0.0184 (9)	0.0187 (9)	0.0282 (10)	0.0007 (8)	−0.0021 (8)	0.0015 (8)
C6A	0.0193 (9)	0.0202 (10)	0.0354 (12)	0.0022 (8)	0.0051 (9)	−0.0005 (8)
C7A	0.0254 (10)	0.0195 (9)	0.0253 (10)	−0.0012 (8)	0.0066 (9)	−0.0002 (8)
C8A	0.0239 (10)	0.0193 (9)	0.0222 (9)	0.0004 (8)	0.0014 (8)	0.0012 (7)
C9A	0.0179 (9)	0.0143 (8)	0.0219 (9)	0.0005 (7)	0.0003 (7)	−0.0012 (7)
C10A	0.0232 (10)	0.0211 (9)	0.0193 (9)	−0.0003 (8)	−0.0058 (8)	−0.0004 (7)
C11A	0.0275 (11)	0.0226 (10)	0.0241 (10)	0.0020 (9)	−0.0021 (9)	0.0022 (8)
S2B	0.0200 (2)	0.0253 (2)	0.0222 (2)	0.00355 (19)	0.00136 (18)	0.00493 (18)
N1B	0.0196 (8)	0.0205 (8)	0.0203 (8)	0.0035 (7)	−0.0015 (7)	0.0033 (6)
N3B	0.0190 (7)	0.0164 (7)	0.0210 (8)	0.0012 (6)	−0.0031 (7)	0.0015 (6)
C2B	0.0185 (9)	0.0152 (8)	0.0229 (9)	0.0000 (7)	−0.0046 (8)	0.0017 (7)
C4B	0.0202 (9)	0.0144 (8)	0.0221 (9)	−0.0014 (8)	−0.0016 (8)	0.0007 (7)
C5B	0.0220 (10)	0.0174 (9)	0.0294 (11)	0.0027 (8)	−0.0034 (9)	0.0010 (8)
C6B	0.0235 (10)	0.0192 (9)	0.0351 (12)	0.0030 (8)	0.0034 (9)	−0.0019 (8)
C7B	0.0288 (10)	0.0191 (9)	0.0263 (10)	−0.0014 (8)	0.0046 (9)	−0.0025 (8)
C8B	0.0261 (10)	0.0174 (9)	0.0219 (9)	−0.0017 (8)	−0.0006 (8)	0.0009 (7)
C9B	0.0197 (9)	0.0144 (8)	0.0231 (9)	−0.0008 (7)	−0.0017 (8)	−0.0003 (7)
C10B	0.0220 (9)	0.0188 (9)	0.0206 (9)	−0.0005 (8)	−0.0037 (8)	0.0044 (7)
C11B	0.0286 (11)	0.0259 (10)	0.0241 (10)	−0.0033 (9)	−0.0066 (9)	0.0001 (8)

### 1-Ethyl-1,3-dihydro-2H-benzo[d]imidazole-2-thione Geometric parameters (Å, º)

**Table:**

S2A—C2A	1.686 (2)	S2B—C2B	1.687 (2)
N1A—C2A	1.356 (3)	N1B—C2B	1.355 (3)
N1A—C9A	1.385 (3)	N1B—C9B	1.385 (3)
N1A—H1A	0.88 (3)	N1B—H1B	0.84 (3)
N3A—C2A	1.365 (3)	N3B—C2B	1.366 (3)
N3A—C4A	1.393 (3)	N3B—C4B	1.392 (3)
N3A—C10A	1.465 (2)	N3B—C10B	1.460 (2)
C4A—C5A	1.391 (3)	C4B—C5B	1.391 (3)
C4A—C9A	1.394 (3)	C4B—C9B	1.399 (3)
C5A—H5A	0.9500	C5B—H5B	0.9500
C5A—C6A	1.390 (3)	C5B—C6B	1.388 (3)
C6A—H6A	0.9500	C6B—H6B	0.9500
C6A—C7A	1.403 (3)	C6B—C7B	1.398 (3)
C7A—H7A	0.9500	C7B—H7B	0.9500
C7A—C8A	1.388 (3)	C7B—C8B	1.388 (3)
C8A—H8A	0.9500	C8B—H8B	0.9500
C8A—C9A	1.388 (3)	C8B—C9B	1.389 (3)
C10A—H10A	0.9900	C10B—H10C	0.9900
C10A—H10B	0.9900	C10B—H10D	0.9900
C10A—C11A	1.516 (3)	C10B—C11B	1.516 (3)
C11A—H11A	0.9800	C11B—H11D	0.9800
C11A—H11B	0.9800	C11B—H11E	0.9800
C11A—H11C	0.9800	C11B—H11F	0.9800
			
C2A—N1A—C9A	110.32 (17)	C2B—N1B—C9B	110.45 (17)
C2A—N1A—H1A	125.1 (19)	C2B—N1B—H1B	125 (2)
C9A—N1A—H1A	123.7 (19)	C9B—N1B—H1B	125 (2)
C2A—N3A—C4A	109.50 (16)	C2B—N3B—C4B	109.61 (16)
C2A—N3A—C10A	124.42 (16)	C2B—N3B—C10B	124.51 (18)
C4A—N3A—C10A	125.52 (17)	C4B—N3B—C10B	125.87 (17)
N1A—C2A—S2A	126.93 (16)	N1B—C2B—S2B	127.00 (15)
N1A—C2A—N3A	106.97 (17)	N1B—C2B—N3B	106.95 (18)
N3A—C2A—S2A	126.10 (15)	N3B—C2B—S2B	126.05 (16)
N3A—C4A—C9A	106.69 (17)	N3B—C4B—C9B	106.57 (17)
C5A—C4A—N3A	131.55 (19)	C5B—C4B—N3B	131.94 (19)
C5A—C4A—C9A	121.76 (19)	C5B—C4B—C9B	121.5 (2)
C4A—C5A—H5A	121.7	C4B—C5B—H5B	121.6
C6A—C5A—C4A	116.6 (2)	C6B—C5B—C4B	116.9 (2)
C6A—C5A—H5A	121.7	C6B—C5B—H5B	121.6
C5A—C6A—H6A	119.2	C5B—C6B—H6B	119.2
C5A—C6A—C7A	121.6 (2)	C5B—C6B—C7B	121.5 (2)
C7A—C6A—H6A	119.2	C7B—C6B—H6B	119.2
C6A—C7A—H7A	119.2	C6B—C7B—H7B	119.2
C8A—C7A—C6A	121.6 (2)	C8B—C7B—C6B	121.7 (2)
C8A—C7A—H7A	119.2	C8B—C7B—H7B	119.2
C7A—C8A—H8A	121.7	C7B—C8B—H8B	121.6
C7A—C8A—C9A	116.66 (19)	C7B—C8B—C9B	116.77 (19)
C9A—C8A—H8A	121.7	C9B—C8B—H8B	121.6
N1A—C9A—C4A	106.50 (17)	N1B—C9B—C4B	106.37 (18)
N1A—C9A—C8A	131.66 (19)	N1B—C9B—C8B	131.99 (19)
C8A—C9A—C4A	121.84 (18)	C8B—C9B—C4B	121.64 (19)
N3A—C10A—H10A	108.9	N3B—C10B—H10C	109.2
N3A—C10A—H10B	108.9	N3B—C10B—H10D	109.2
N3A—C10A—C11A	113.41 (17)	N3B—C10B—C11B	112.01 (17)
H10A—C10A—H10B	107.7	H10C—C10B—H10D	107.9
C11A—C10A—H10A	108.9	C11B—C10B—H10C	109.2
C11A—C10A—H10B	108.9	C11B—C10B—H10D	109.2
C10A—C11A—H11A	109.5	C10B—C11B—H11D	109.5
C10A—C11A—H11B	109.5	C10B—C11B—H11E	109.5
C10A—C11A—H11C	109.5	C10B—C11B—H11F	109.5
H11A—C11A—H11B	109.5	H11D—C11B—H11E	109.5
H11A—C11A—H11C	109.5	H11D—C11B—H11F	109.5
H11B—C11A—H11C	109.5	H11E—C11B—H11F	109.5
			
N3A—C4A—C5A—C6A	−179.6 (2)	N3B—C4B—C5B—C6B	179.4 (2)
N3A—C4A—C9A—N1A	−1.1 (2)	N3B—C4B—C9B—N1B	−0.7 (2)
N3A—C4A—C9A—C8A	179.75 (18)	N3B—C4B—C9B—C8B	179.23 (18)
C2A—N1A—C9A—C4A	1.1 (2)	C2B—N1B—C9B—C4B	−0.8 (2)
C2A—N1A—C9A—C8A	−179.9 (2)	C2B—N1B—C9B—C8B	179.3 (2)
C2A—N3A—C4A—C5A	−178.9 (2)	C2B—N3B—C4B—C5B	−176.8 (2)
C2A—N3A—C4A—C9A	0.8 (2)	C2B—N3B—C4B—C9B	1.9 (2)
C2A—N3A—C10A—C11A	91.4 (2)	C2B—N3B—C10B—C11B	94.4 (2)
C4A—N3A—C2A—S2A	179.34 (14)	C4B—N3B—C2B—S2B	176.59 (15)
C4A—N3A—C2A—N1A	−0.2 (2)	C4B—N3B—C2B—N1B	−2.3 (2)
C4A—N3A—C10A—C11A	−98.0 (2)	C4B—N3B—C10B—C11B	−84.5 (2)
C4A—C5A—C6A—C7A	−0.4 (3)	C4B—C5B—C6B—C7B	0.3 (3)
C5A—C4A—C9A—N1A	178.60 (17)	C5B—C4B—C9B—N1B	178.17 (18)
C5A—C4A—C9A—C8A	−0.5 (3)	C5B—C4B—C9B—C8B	−1.9 (3)
C5A—C6A—C7A—C8A	−0.2 (3)	C5B—C6B—C7B—C8B	−0.5 (3)
C6A—C7A—C8A—C9A	0.4 (3)	C6B—C7B—C8B—C9B	−0.5 (3)
C7A—C8A—C9A—N1A	−178.9 (2)	C7B—C8B—C9B—N1B	−178.5 (2)
C7A—C8A—C9A—C4A	−0.1 (3)	C7B—C8B—C9B—C4B	1.7 (3)
C9A—N1A—C2A—S2A	179.93 (14)	C9B—N1B—C2B—S2B	−176.99 (15)
C9A—N1A—C2A—N3A	−0.6 (2)	C9B—N1B—C2B—N3B	1.9 (2)
C9A—C4A—C5A—C6A	0.7 (3)	C9B—C4B—C5B—C6B	0.9 (3)
C10A—N3A—C2A—S2A	−8.8 (3)	C10B—N3B—C2B—S2B	−2.5 (3)
C10A—N3A—C2A—N1A	171.69 (17)	C10B—N3B—C2B—N1B	178.59 (18)
C10A—N3A—C4A—C5A	9.4 (3)	C10B—N3B—C4B—C5B	2.3 (3)
C10A—N3A—C4A—C9A	−170.92 (17)	C10B—N3B—C4B—C9B	−179.08 (18)

### 1-Ethyl-1,3-dihydro-2H-benzo[d]imidazole-2-thione Hydrogen-bond geometry (Å, º)

Cg3 is the centroid of the C4A···C9A ring.

**Table:**

*D*—H···*A*	*D*—H	H···*A*	*D*···*A*	*D*—H···*A*
N1*B*—H1*B*···S2*A*^i^	0.84 (3)	2.49 (3)	3.3258 (18)	171 (3)
N1*A*—H1*A*···S2*B*^ii^	0.88 (3)	2.41 (3)	3.2654 (18)	165 (3)
C6*B*—H6*B*···*Cg*3^iii^	0.95	2.85	3.6470 (17)	143

Symmetry codes: (i) *x*+1, *y*, *z*; (ii) *x*−1, *y*, *z*; (iii) *x*+3/2, −*y*−1/2, −*z*.
